# *MicroRNA-124-3p* inhibited progression of nasopharyngeal carcinoma by interaction with PCDH8 and the inactivation of PI3K/AKT/mTOR pathway

**DOI:** 10.7150/jca.57152

**Published:** 2021-06-11

**Authors:** Jiacai Ye, Quanxing Liao, Xiaohui Zeng, Chang Liu, Yan Ding, Xuefeng Liu, Lisi Zeng, Tianpei Guan, Yawei Yuan

**Affiliations:** 1 Department of Radiation Oncology, Nanfang Hospital, Southern Medical University, Guangzhou, China.; 2 Department of Radiation Oncology, Affiliated Cancer Hospital and Institute of Guangzhou Medical University, Guangzhou, China.; 3 Institute of Oncology, Affiliated Cancer Hospital & Institute of Guangzhou Medical University, Guangzhou, China.; 4 Department of Abdominal Surgery (Section 2), Affiliated Cancer Hospital & Institute of Guangzhou Medical University, Guangzhou, China.

**Keywords:** *MicroRNA-124-3p*, tumor proliferation, growth, nasopharyngeal carcinoma, protocadherin-8, phosphatidylinositol 3-kinase/AKT/mammalian target of rapamycin

## Abstract

Nasopharyngeal carcinoma (NPC) is characterised by distinct geographical distribution and is particularly prevalent in Asian countries. But the mechanisms related to the progression of nasopharyngeal carcinoma (NPC) are not completely understood. MiR-124-3p functions as a tumor suppressor in many kinds of human cancers. Here, we explored the effects and mechanism of *miR-124-3p* on the proliferation and colony formation in NPC. In our study, we reported that miR-124-3p was significantly downregulated in NPC tissues and cell lines*.* Overexpression *miR-124-3p* decreased NPC cell proliferation and colony formation abilities. Meanwhile, knockdown *miR-124-3p* increased proliferation and colony formation abilities. Additionally, dual-luciferase assay showed that miR-124-3p could positively regulated PCDH8 by targeting its 3'-UTR. Overexpression of PCDH8 could partially rescue the proliferation and colony formation role of *miR-124-3p* inhibitor. Our study indicated that *miR-124-3p* played a tumor suppressor by directly interacting with PCDH8 and inhibiting the activation of the phosphatidylinositol 3-kinase (PI3K)/AKT/mammalian target of rapamycin (mTOR) signaling pathway. Overall, we found that *miR-124-3p* inhibited the activation of the PI3K/AKT/mTOR signaling pathway in NPC by interacting with PCDH8. Thus, PCDH8 may be a potential molecular target that impeded NPC proliferation and colony formation.

## Introduction

Nasopharyngeal carcinoma (NPC) is a malignant type of head and neck squamous cell carcinoma occurring in epithelial cells of the nasopharynx 1, 2. NPC is associated with distinct geographical distribution and racial differences, and it is highly prevalent in Asian countries 3, 4. NPC is prone to invasion and distant metastasis at an early stage, and more than 70% of patients are diagnosed with advanced NPC during their initial assessment 5. NPC is sensitive to radiotherapy; thus, this treatment strategy has greatly improved local control of lesions and the 5-year survival rate in patients with NPC 5-8. However, frequent local recurrence, metastasis, and poor prognosis remain major challenges in the clinical treatment of NPC because the mechanisms related to NPC progression are not completely understood 9-11.

MicroRNAs (miRNAs) play critical roles as oncogenes or tumor suppressors in NPC. MiRNAs are a category of small non-coding RNAs (~19-25 nucleotides) that regulate essential biological processes by directly interacting with their target mRNAs at 3′-untranslated regions (3′-UTRs) 12-15. Recent studies have reported that multiple miRNAs (e.g., *miR-26a*, *miR-101*, *miR-98*, and *miR-214*) are abnormally expressed in patients with NPC and contribute to the development and progression of NPC 16-19. *MiR-124-3p* is the mature sequence of *miR-124* in humans and acts as a tumor suppressor in some cancer types, such as esophageal squamous cell carcinoma and glioma 20, 21. Furthermore, *miR-124* played essential roles in the development and progression of NPC 22-27. Xu et al. demonstrated that NPC growth and metastasis were inhibited by the overexpression of *miR-124-3p* 28. Nevertheless, the potential function and mechanism of tumor suppression remain unclear.

Protocadherin-8 (PCDH8) is predicted to be a target of *miR-214-3p* using targetscan (http://www.targetscan.org). PCDH8 is an integral membrane protein that acts as a tumor suppressor in several types of cancer 29-31 and is involved in the metastasis of gastric cancer 32. Additionally, PCDH8 has been found to be regulated by *miR-217*, which promotes apoptosis in small cell lung cancer 33. PCDH8 also plays tumor-suppressive roles in NPC 34.

In this study, function assays found that overexpression of *miR-124-3p* decreased NPC cell proliferation and colony formation abilities, meanwhile knockdown of *miR-124-3p* increased proliferation and colony formation abilities. Mechanism assays indicated that *miR-124-3p* played a tumor suppressor by directly interacting with PCDH8 and inhibiting the activation of PI3K/AKT/mTOR signaling pathway.

## Materials and Methods

### Cell culture

The human nasopharyngeal epithelial cell line NP69 and four human NPC cell lines (CNE2, 6-10B, S18, and HK1) were obtained from Guangzhou Medical University Surgical Laboratory (Guangzhou, China) and cultured in Roswell Park Memorial Institute-1640 (RPMI-1640; Gibco, Rockville, MD, USA) medium containing 10% fetal bovine serum (Gibco Life Technologies, Rockville, MD, USA) and 1% penicillin/streptomycin (Gibco). All cell lines were cultured at 37°C with 5% carbon dioxide.

### Cell transfection

*Hsa-miR124-3p* mimics and inhibitor lentiviral packaging constructs were obtained from GenePharma Biotechnology Co., Ltd. (Shanghai, China). For cell transfection, cells were seeded into six-well plates, and lentivirus (50 µL “*lv-mir124-3p* mimic” or 50 µL “*lv-mir124-3p* inhibitor”) and 3 µL polybrene were added to each well. Puromycin (2.0 mg/mL) was then added to each well, and plates were incubated for 14 days to select cells with stable expression of constructs. Cells infected with lentiviral nonsense scramble control miRNA were used as the control.

### RNA isolation and quantitative polymerase chain reaction (PCR)

Total RNA from NPC cell lines and tumor tissue from xenograft were extracted from cells using TRIzol reagent (Invitrogen, Carlsbad, CA, USA). The quality and concentration of total RNA were measured using a NanoDrop 2000 spectrophotometer (Thermo Scientific, USA). The GoScript Reverse Transcription System (Promega, Madison, WI, USA) was used for cDNA synthesis from 2 μg of total RNA. Real-time quantitative PCR was performed with 2 μL cDNA template and 7.5 μL GoTaq quantitative PCR Master Mix (Promega) in a 15-μL total reaction volume. Samples were processed on a CFX96 Touch Real-Time PCR Detection system (Bio-Rad Laboratories, Hercules, CA, USA) using the manufacturer's protocol. Primers were synthetized by Shanghai Thermo Fisher Company (Shanghai, China). Glyceraldehyde 3-phosphate dehydrogenase (*GAPDH*) and *U6* genes were used as internal standards. The following primers were used: *GAPDH* forward primer, 5′-CTCCTCCTGTTCGACAGTCAGC-3′; *GAPDH* reverse primer, 5′-CCCAATACGACCAAATCCGTT-3′; *U6* forward primer, 5′-CTCGCTTCGGCAGCACA-3′; *U6* reverse primer, 5′-AACGCTTCACGAATGCGT-3′; *miR-124-3p* forward primer, 5′-CTCAACTGGTGTCGTGGAGTCGGCAATTCAGTTGAGGGCATTCA-3′; *miR-124-3p* reverse primer, 5′-ACACTCCAGCTGGGTAAGGCACGCGGTGAATGCC-3′; *PCDH8* forward primer, 5′-TATGGGCACGAGCACTTCC-3′; *PCDH8* reverse primer, 5′-CCAGCGTCAAGTTGTACTCGG-3′. All experiments were performed in triplicate and relative expression was calculated using the 2^-ΔΔCt^ method.

### Cell Counting Kit-8 (CCK-8) assay

The proliferative activity of cells was detected by CCK-8 assays (Dojindo Laboratories, Kumamoto, Japan). NPC cells were collected during the logarithmic phase, and then cell suspension was incubated in a 96-well plate (3 ×10^3^ cells/well) at 37°C in 5% CO2 overnight, until all cells adhered to the wall.After cell attachment, transfection was performed using Lipofectamine 3000 and P3000 (Invitrogen) according to the manufacturers instructions. After transfection for 0, 24, 48, 72, 96, or 120 h, 10 μL CCK-8 solution was added to each well, and the cells were incubated for 2 h at 37°C. The absorbance of each well was detected at 450 nm using a multifunctional microplate reader.

### Colony formation assay

The colony-forming activity of cells was examined by colony formation assay. NPC cells of logarithmic growth phase were collected and plated into 12-well plates (100 cells/well. All cells were cultured and maintained at 37°C in an incubator with 5% CO2. Twelve days after seeding, the cells were washed with *PBS* thrice and fixed with methanol for 10 min. After fixation, cells were washed three times with PBS. Then the colonies containing more than 50 cells was counted after staining with crystal violet.

### Western blotting

Lysis buffer containing a protease inhibitor was used for processing NPC cell protein extracts. The protein concentration of each sample was measured with a bicinchoninic acid protein assay kit (KenGEN BioTECH, Nanjing, China). First, 30 μg of each protein sample was separated using sodium dodecyl sulfate-polyacrylamide gel electrophoresis and transferred to polyvinylidene difluoride membranes. The membranes were blocked with 5% skimmed milk for 2 h at room temperature and incubated with primary antibodies at 4°C overnight. The primary antibodies were used for western blotting (Supplementary table S1). The membranes were washed three times with Tris-buffered saline and Tween-20 and then incubated at room temperature for 2 h with horseradish peroxidase-conjugated secondary antibodies (anti-rabbit or anti-mouse; Cell Signaling Technology). β-Tubulin was used as an endogenous control to compare the protein expression. A chemiluminescence system (Tanon 5200; Tanon Science & Technology Co., Ltd., Shanghai, China) was used to visualize the proteins on the membranes.

### Dual-luciferase reporter assay

Cells were seeded into 24-well plates to examine the relationships between *miR-124-3p* and PCDH8. Cells were cotransfected with 200 ng PCDH8 (wild-type [WT]) or PCDH8 (mutant [MUT]) reporter plasmids and 20 nM miR-NC or *miR-124* with Lipofectamine 3000 reagent (Invitrogen). The firefly and Renilla luciferase activities were measured using a dual-Glo luciferase reporter assay kit (Promega).

### Tumor growth in xenograft model

All animal experiments were conducted under protocols approved by the Animal Experimentation Ethics Committee of Guangzhou Medical University (No.2005DKA21300). 6~8-week old immunodeficient BABL/c nude mice were used assay as a xenograft model for tumor growth. Cells were counted (5×10^6^) and injected into the right dorsal flank of BABL/c nude mice. The weight of mice and volume of tumor were measured every 4 days, tumor volume was calculated using the formula: Tumor maximum diameter (L) × the right-angle diameter to that axis (W)^2^/2. After 4 weeks of injection, mice were sacrificed according to institutional ethical guidelines, the xenograft tumors were dissected and weighted comparing to control groups *in vivo*.

### *In situ* hybridization (ISH)

*In situ* hybridization was performed using single-strand RNA probes comprising antisense or sense sequences of decorin or biglycan. Digoxigenin (DIG)-labeled RNA probes were synthesized by *in vitro* transcription with microRNA ISH Buffer using a DIG RNA Labeling Kit (Roche). Chip HLugA180Su01 were fixed and hybridized with RNA probes. Hybridization signals were detected immunohistochemically by anti-DIG-AP Fab fragments. The reagents used are shown in Supplementary table S2.

### Immunohistochemistry (IHC)

IHC for target molecules (PCDH8) was performed on sections from tumor tissues of nude mice xenografts. Tissue sections were deparaffinized, subjected to antigen retrieval using 0.01M sodium citrate solution, and incubated with immunohistochemical ultrasensitive kit (MXB Biotechnologies, Fuzhou, China) and primary antibodies against PCDH8. Then sections were incubated with 3,3-Diaminobenzidine (DAB). Quantification of PCDH8 was measured by intensity and extent of staining.

### Statistical analysis

Data were analyzed using Statistical Product and Service Solutions version 20.0 (IBM Corp., Armonk, NY, USA). The differences between two groups were compared with Student's *t*-tests or Pearson's χ^2^-tests. One-way analysis of variance was used for comparisons of multiple groups. P value of <0.05 was considered as a statistically significant difference.

## Results

### *miR-124-3p* was expressed at low levels in NPC tissues and cell lines

**Figure 1:**
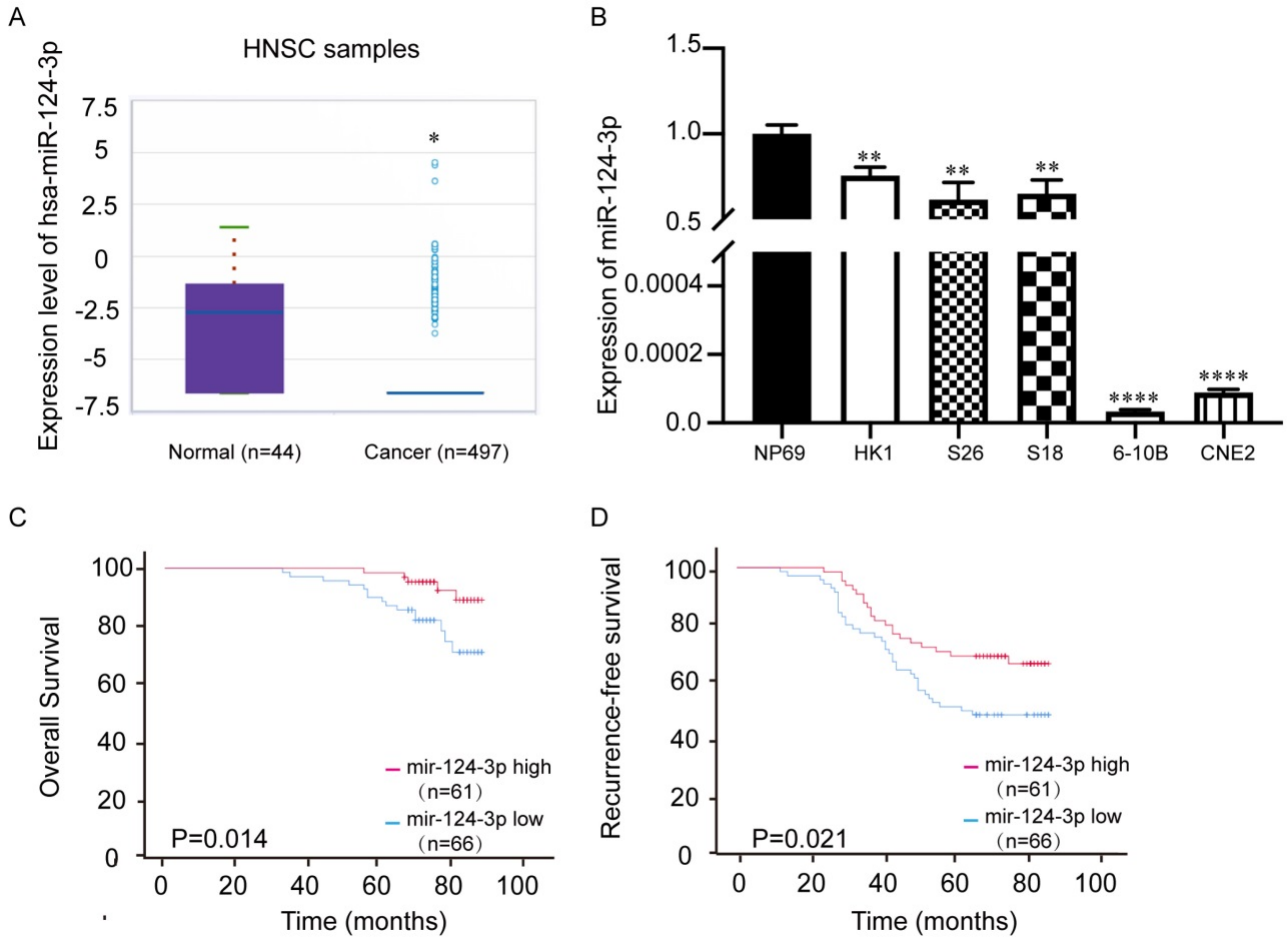
*miR-124-3p* expression was decreased in nasopharyngeal carcinoma tissues and cell lines compared with that in normal tissues. (A) Expression of *miR-124-3p* in head and neck squamous cell carcinoma compared to that in healthy tissues. (B) Expression of *miR-124-3p* in CNE2, 6-10B, S18, and HK1 cells. (C) Overall survival of nasopharyngeal carcinoma (NPC) patients with high or low *miR-124-3p*. (D) Recurrence-free survival (RFS) of NPC patients with high or low *miR-124-3p*. Higher *miR-124-3p* expression was associated with higher OS and RFS.

The miRbase website (http://mirbase.org/) showed that the expression of *miR-124-3p* in head and neck squamous cell carcinoma tissues was significantly downregulated (Fig. 1A), which indicated *miR-124-3p* may play a tumor suppressor role in tumorigenesis. To further evaluate the expression of *miR-124-3p*, qPCR showed that the expression of *miR-124-3p* was significantly downregulated in NPC cell lines, especially 6-10B and CNE2 cell lines (Fig. 1B). To further explore the importance of miR-124-3p in clinically, NPC tissue microarray (Chip HLugA180Su01) was used *in situ* hybridization. In order to explore the relationship between miR-124-3p and clinical parameters of NPC patients, NPC patients were divided into two groups: high expression group and low expression group according to the median expression of miR-124-3p. The correlation between the expression of miR-124-3p and clinicopathological data was analyzed. We analyzed the effect of high and low expression of miR-124-3p on the overall survival (OS) and recurrence-free survival (RFS) in patients with NPC. The results showed that compared with patients with lower expression of miR-124-3p, NPC patients with higher expression of miR-124-3p had significantly longer 5-year OS and 5-year RFS (Fig. 1 C and D). Cox and multivariate cox regression analysis showed that miR-124-3p, clinical stage was correlated with prognosis (Table 1, Table 2 and 2A).

### Upregulation of *miR-124-3p* decreased proliferation and colony formation in NPC cells

**Figure 2:**
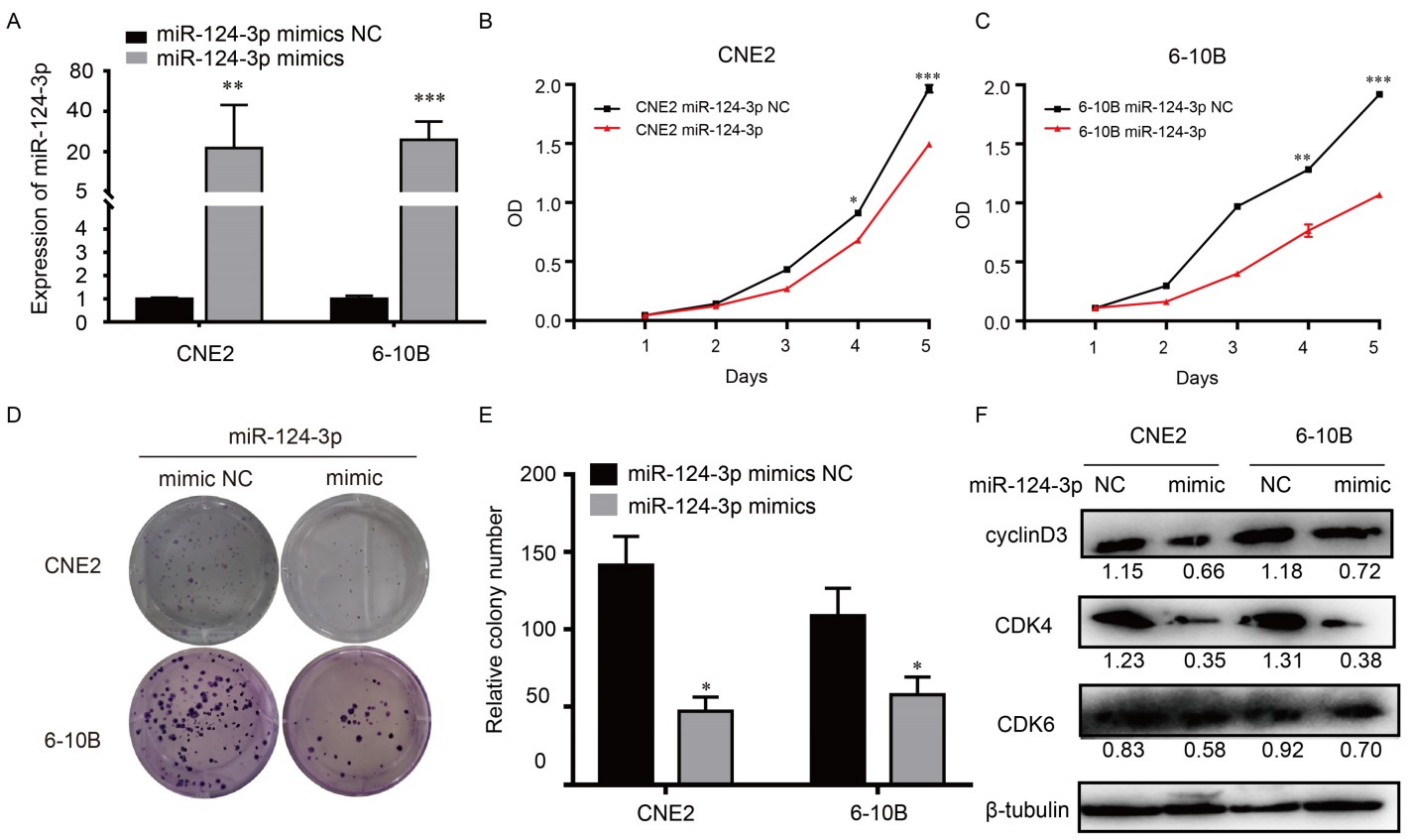
Upregulation of *miR-124-3p* expression decreased cell proliferation and colony formation in nasopharyngeal carcinoma cells. (A) Effects of miR-124-3p mimic on miR-124-3p expression in CNE2 and 6-10B cells. (B and C) Effects of *miR-124-3p* overexpression on CNE2 and 6-10B cell proliferation, as evaluated using CCK-8 assays. (D and E) Effects of *miR-124-3p* overexpression on colony formation in CNE2 and 6-10B cells, as evaluated using plate cloning. (F) Effects of *miR-124-3p* overexpression on cyclin D3 and CDK4 expression in CNE2 and 6-10B cells, as determined by western blotting.

To explore the potential role of *miR-124-3p* in NPC, cells were transfected with *miR-124-3p* mimic, accordingly, *miR-124-3p* could be upregulated in 6-10B and CNE2 cells (Fig. 2A). To know the function of miR-124-3p, function assays were further explored. As shown in Fig. 2B and 2C, after overexpression of miR-124-3p, cell proliferation were significantly decreased in the miR-124-3p mimic group compared with those in the mimic negative control (NC) group (P < 0.05). Similarly, after overexpression of miR-124-3p, colony formation was significantly decreased in the miR-124-3p mimic group compared with those in the mimic negative control (NC) group (P < 0.05; Fig. 2D and 2E). To further evaluation the role of miR-124-3p expression on proliferation, western blotting was used to detect the proliferation related protein. As shown in Fig. 2F, overexpression of miR-124-3p in CNE2 and 6-10B cells caused a decrease in cyclin D3 and cyclin-dependent kinase 4 protein levels (CDK4). Thus, these results indicated that miR-124-3p affected cell progression by regulating the expression of cyclin D3 and CDK4.

### Knockdown *miR-124-3p* increased proliferation and colony formation in NPC cells

**Figure 3:**
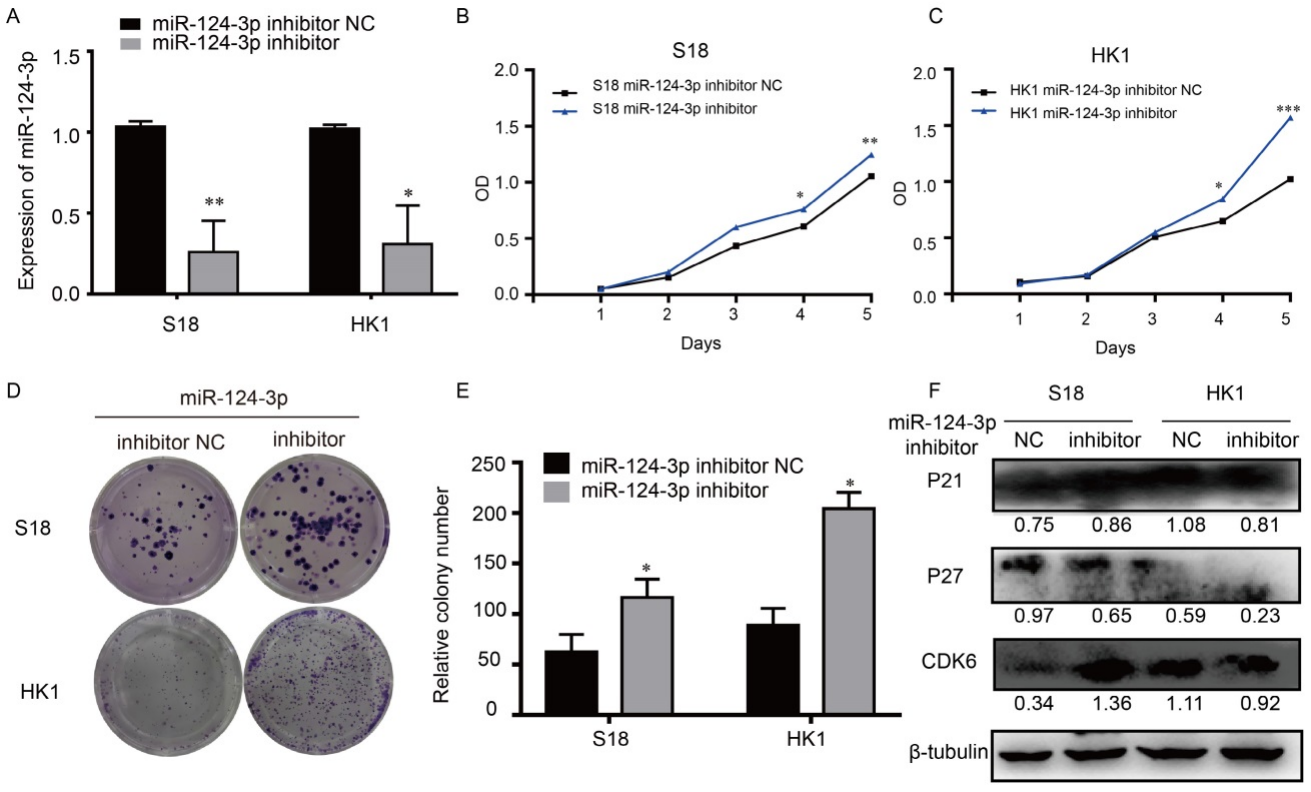
Knockdown *miR-124-3p* expression increased cell proliferation and colony formation in nasopharyngeal carcinoma cells. (A) Effects of miR-124-3p inhibitor on miR-124-3p expression in S18 and HK1 cells. (B and C) Effects of *miR-124-3p* down expression on S18 and HK1 cell proliferation, as evaluated using CCK-8 assays. (D and E) Effects of *miR-124-3p* down expression on colony formation in S18 and HK1 cells, as evaluated using plate cloning. (F) Effects of *miR-124-3p* down expression on P21, P27 and CDK6 expression in S18 and HK1 cells, as determined by western blotting.

In similar opposite way, to explore the potential role of *miR-124-3p* in NPC, cells were transfected with *miR-124-3p* inhibitor, *miR-124-3p* could also be knockdown in S18 and HK1 cells (Fig. 3A). The function of miR-124-3p assays were also further explored. As shown in Fig. 3B and 3C, after down expression of miR-124-3p, cell proliferation were significantly increased in the miR-124-3p mimic group compared with those in the mimic negative control (NC) group (P < 0.05). Similarly, after down expression of miR-124-3p, colony formation were significantly increased in the miR-124-3p inhibitor group compared with those in the mimic negative control (NC) group (P < 0.05; Fig. 3D and 3E). To further evaluation the role of miR-124-3p expression on proliferation, western blotting was used to detect the proliferation related protein. As shown in Fig. 3F, down expression of miR-124-3p in S18 and HK1 cells caused a decrease in P27 and an increase in cyclin-dependent kinase 6 protein levels (CDK6). Thus, these results indicated that miR-124-3p affected cell progression by regulating the expression of P27 and CDK6.

### *miR-124-3p* positively regulated PCDH8 by targeting its 3'-UTR

**Figure 4:**
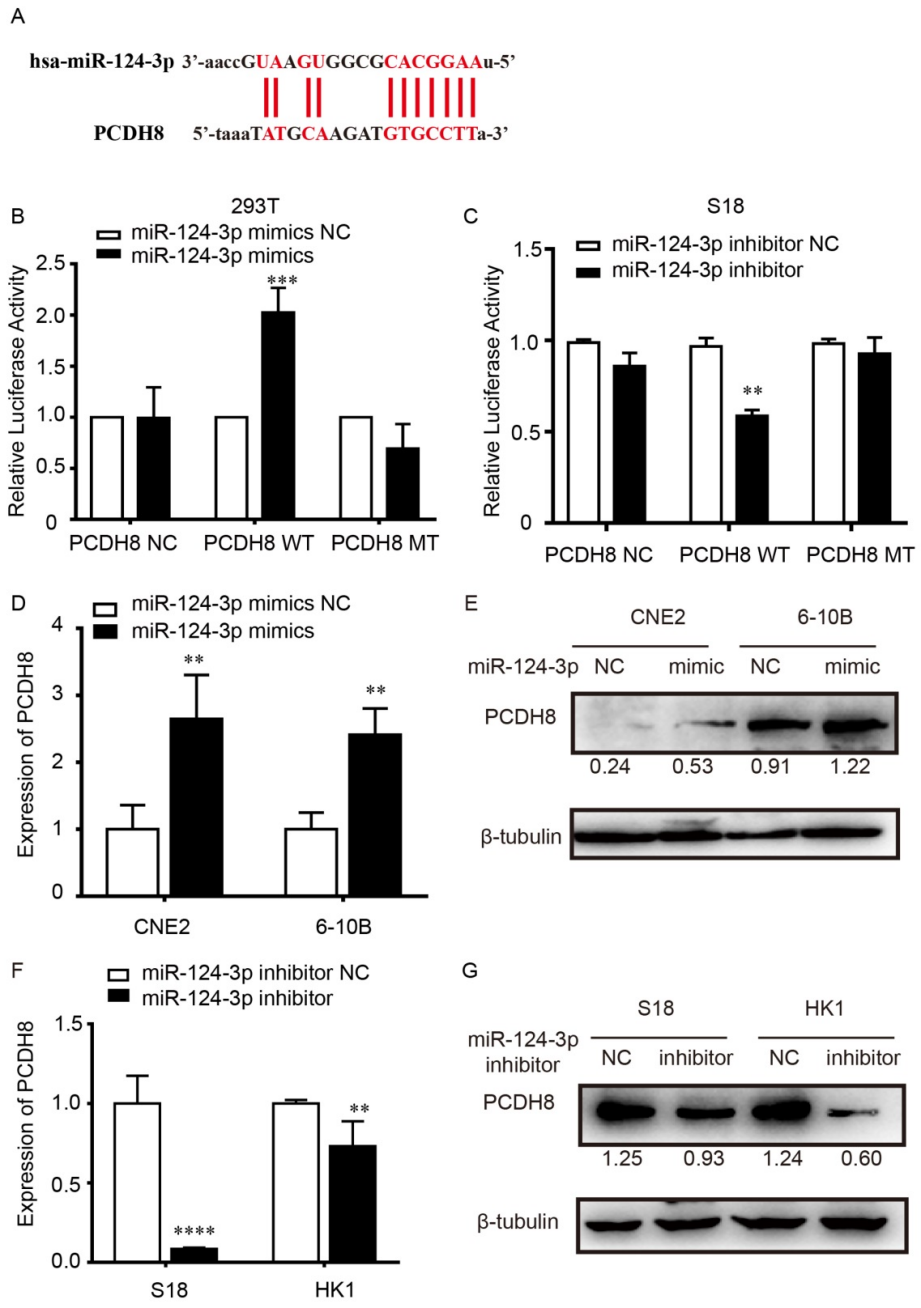
*miR-124-3p* positively regulated *PCDH8* by interacting with its 3′-UTR. (A) The 3′-UTR of *PCDH8* was a binding target for *miR-124-3p.* (B) Effects of *miR-124-3p* overexpression on firefly luciferase activity in 293t cells cotransfected with PCDH8-WT or -MUT. (C) Effects of *miR-124-3p* inhibitor on firefly luciferase activity in S18 cells cotransfected with PCDH8-WT or -MUT. (D and E) Effects of *miR-124-3p* mimic in CNE2 and 6-10B cells on *PCDH8* mRNA expression. (F and G) Effects of *miR-124-3p* inhibitor in S18 and HK1 cells on *PCDH8* mRNA expression.

To explore the potential target of *miR-124-3p,* the target genes of *miR-124-3p* were investigated using TargetScan (http://www.targetscan.org/). The results showed that the 3′-UTR sequence in *PCDH8* may be a binding target of *miR-124-3p* (Fig. 4A). Dual-luciferase assay showed that, compared with that in the NC group, after transfected with the PCDH8-WT plasmid in 293T cells, the firefly luciferase activity of *miR-124-3p* significantly increased. However, cells transfected with the PCDH8-MUT plasmid, the firefly luciferase activity was significantly changed (Fig. 4B). And we found the same phenomenon in S18 cells (Fig. 4C). Moreover, qPCR and western blotting showed that overexpression of *miR-124-3p* significantly increased the mRNA and protein level of PCDH8 (*P* < 0.01; Fig. 4D and 4E), whereas *miR-124-3p* inhibition could decrease the mRNA and protein of PCDH8 (*P* < 0.01; Fig. 4F and 4G). The expression of PCDH8 in CNE2 and 6-10B cells were significantly upregulated in cells transfected with *miR-124-3p* mimic compared with those in the mimic NC group. These results indicated that PCDH8 miR-124-3p, miR-124-3p may suppress the NPC proliferation by regulating *PCDH8* transcriptional activity.

### *PCDH8* rescued tumor suppressor role of *miR-124-3p*

**Figure 5:**
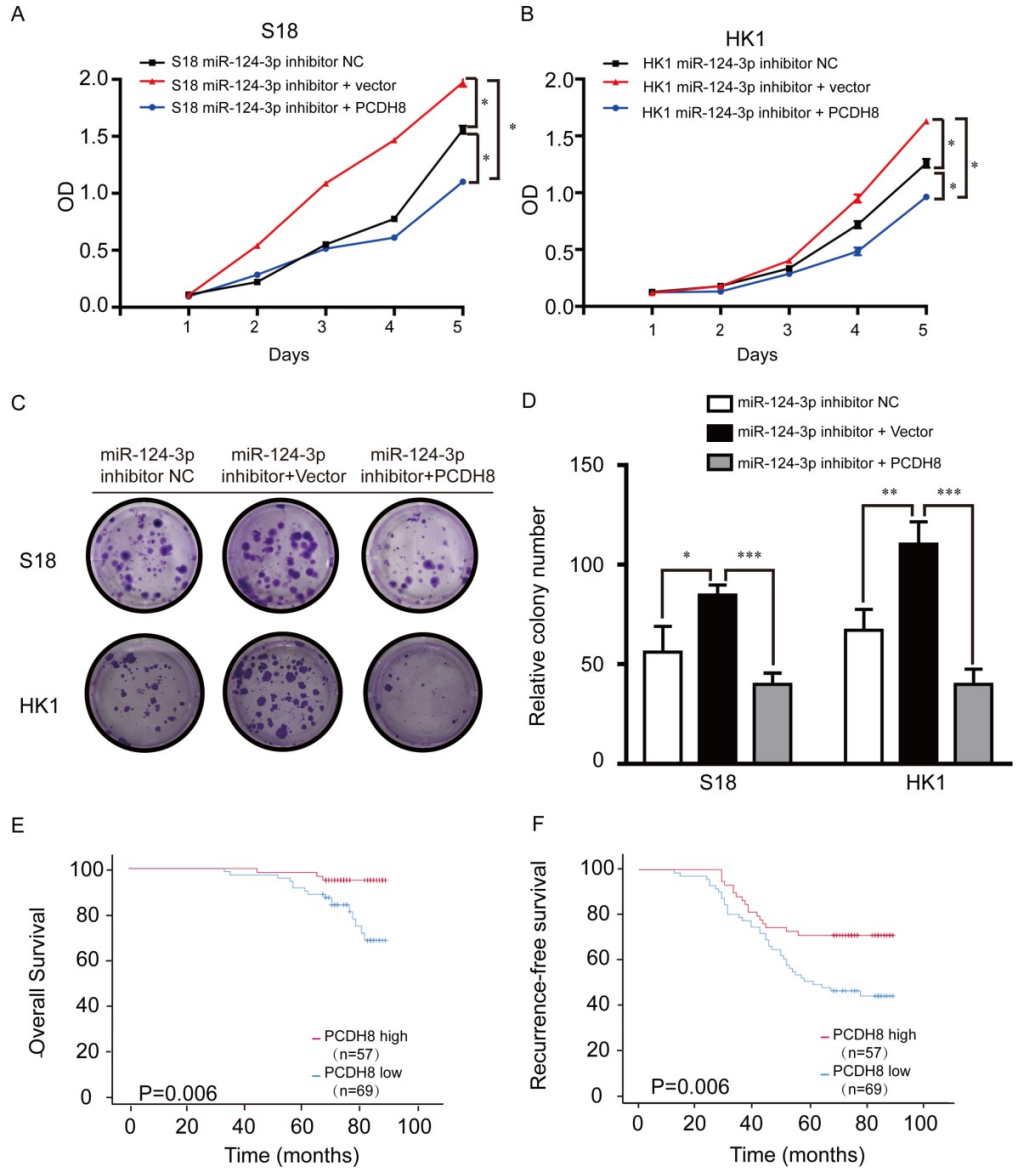
PCDH8 rescued the suppression of *miR-124-3p* in NPC cell lines. (A and B) Effects of *miR-124-3p* inhibitor treatment on cell proliferation in S18 cells (A) and HK1 cells (B). (C and D) Effects of *miR-124-3p* inhibitor on cell proliferation and colony formation. (E) Overall survival of nasopharyngeal carcinoma (NPC) patients with high or low PCDH8. (F) RFS of NPC patients with high or low PCDH8. Higher PCDH8 expression was associated with higher OS and RFS.

PCDH8 plasmids were transfected into NPC cell lines to explore the relationship between PCDH8 expression and *miR-124-3p*. Treatment with *miR-124-3p* inhibitor markedly promoted cell proliferation in S18 (Fig. 5A) and HK1 cells (Fig. 5B) endogenously expressing PCDH8. To determine whether ectopic overexpression of PCDH8 could rescue the suppressive effects of *miR-124-3p* on cell proliferation, *lv-miR-124-3p* inhibitor was transfected into S18 and HK1 cells. We observed that the cell proliferation and colony formation were inhibited in the infected *miR-124-3p* inhibitor and PCDH8 plasmid group compared with those in the inhibitor vector control and inhibitor NC groups. Further, transfection with PCDH8 and *mir-124-3p* inhibitors impaired the ability of PC cell clone formation. (Fig. 5C and D). CCK8 and colony-forming assays showed that PCDH8 could partially reduce the *miR-124-3p* inhibitor-mediated activity in S18 and HK1 cells. To further explore the importance of PCDH8 in clinical survival, NPC tissue microarray (Chip HLugA180Su01) was used in immunohistochemistry. In order to explore the relationship between PCDH8 and clinical parameters of NPC patients, NPC patients were divided into two groups: high expression group and low expression group according to the median expression of PCDH8. The correlation between the expression of PCDH8 and clinicopathological data was analyzed. We analyzed the effect of high and low expression of PCDH8 on the OS and RFS in patients with NPC. The results showed that compared with patients with lower expression of PCDH8, NPC patients with higher expression of PCDH8 had significantly longer 5-year OS and 5-year RFS (Fig. 5 E and F). PCDH8 and clinical parameters were analyzed by cox univariate and multivariate regression analysis to explore the prognostic factors. Clinically regression analysis demonstrated that PCDH8, clinical stage was also correlated with good OS and RFS (Table 3, Table 4 and 4A). Conjointly, these results suggested that PCDH8 could be used as potential biomarker to predict the prognosis of patients with NPC.

### *miR-124-3p* suppress tumor formation of NPC cells in mouse xenograft models

**Figure 6:**
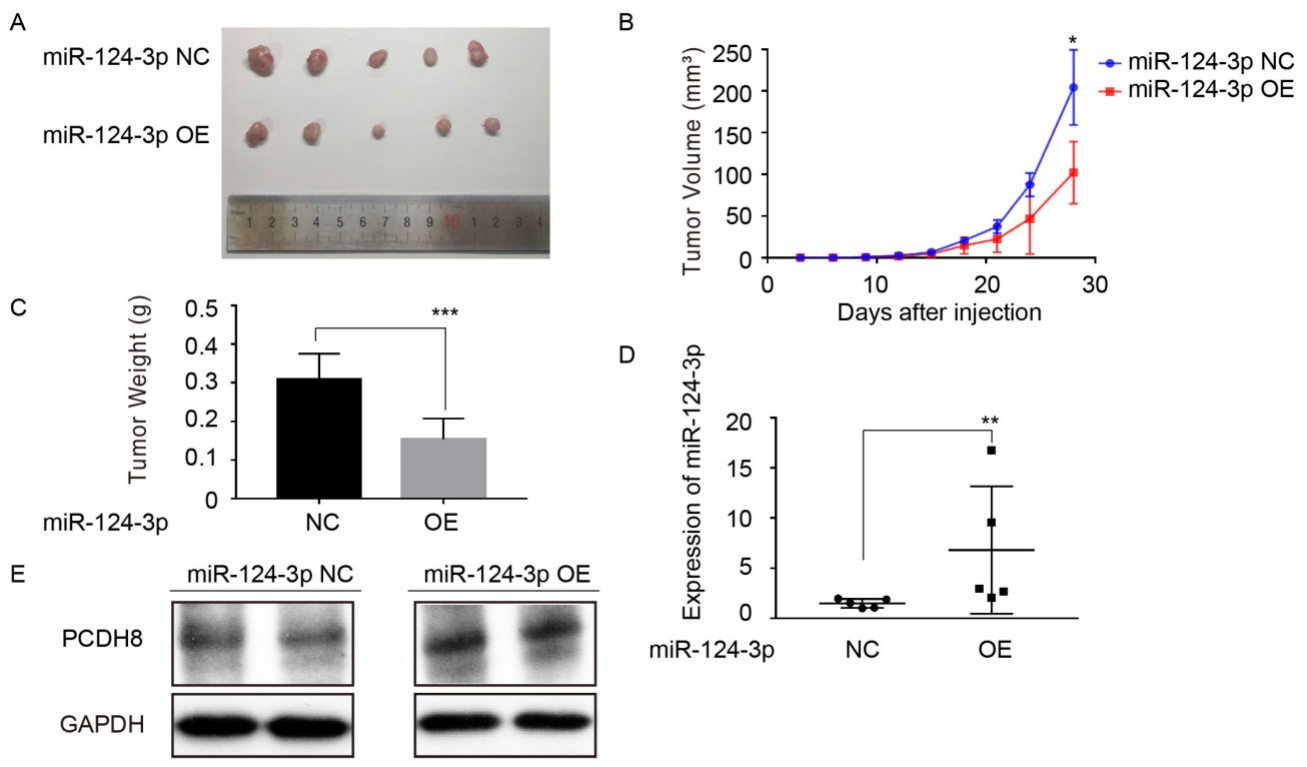
Upregulation of *miR-124-3p* inhibit tumorigenesis of nasopharyngeal carcinoma cells in nude mice. (A) Four weeks after injected with CNE2 cell in subcutaneous on nude mice, the group of overexpressing *miR-124-3p* was significantly smaller than that formed by the CNE2 cells transfected with negative group. (B) The tumor volume of subcutaneously implanted tumor in nude mice with *miR-124-3p* overexpression decreased significantly than negative group. (C) The weight of subcutaneously implanted tumor in nude mice with *miR-124-3p* overexpression decreased significantly than negative group. (D) qPCR showed that the expression of *miR-124-3p* in the subcutaneously transplanted tumor tissue was significantly increased in *miR-124-3p* overexpression group. (E) Western blot analysis indicated that the expression of PCDH8 was decreased in the subcutaneously transplanted tumor tissue in the *miR-124-3p* overexpression group.

To further investigate the effect of miR-124-3p on the proliferation of NPC cells in *vivo*. CNE2 cell with high expression of miR-124-3p were injected into the subcutaneous of nude mice. Compared with the control group, miR-124-3p overexpression resulted in a smaller size of the subcutaneous tumor in mice (Fig. 6A). And the mean tumor volume for miR-124-3p overexpression group was significantly smaller than NC group (Fig. 6B). Additionally, the weight of tumor tissue was lower in the miR-124-3p overexpression group than that of the NC group (Fig. 6C). Moreover, qRT-PCR analysis suggested that miR-124-3p expression was obviously increased in tumor tissue of miR-124-3p overexpressed nude mice. (Fig. 6D), and Western blotting assay showed that the protein of PCDH8 was increased when miR-124-3p overexpressed (Fig. 6E). Collectively, these results above suggested that miR-124-3p suppress proliferation of NPC cell *in vivo*.

### *miR-124-3p* inhibited NPC cell proliferation and growth by inactivating the PI3K/AKT/mTOR signaling pathway

**Figure 7:**
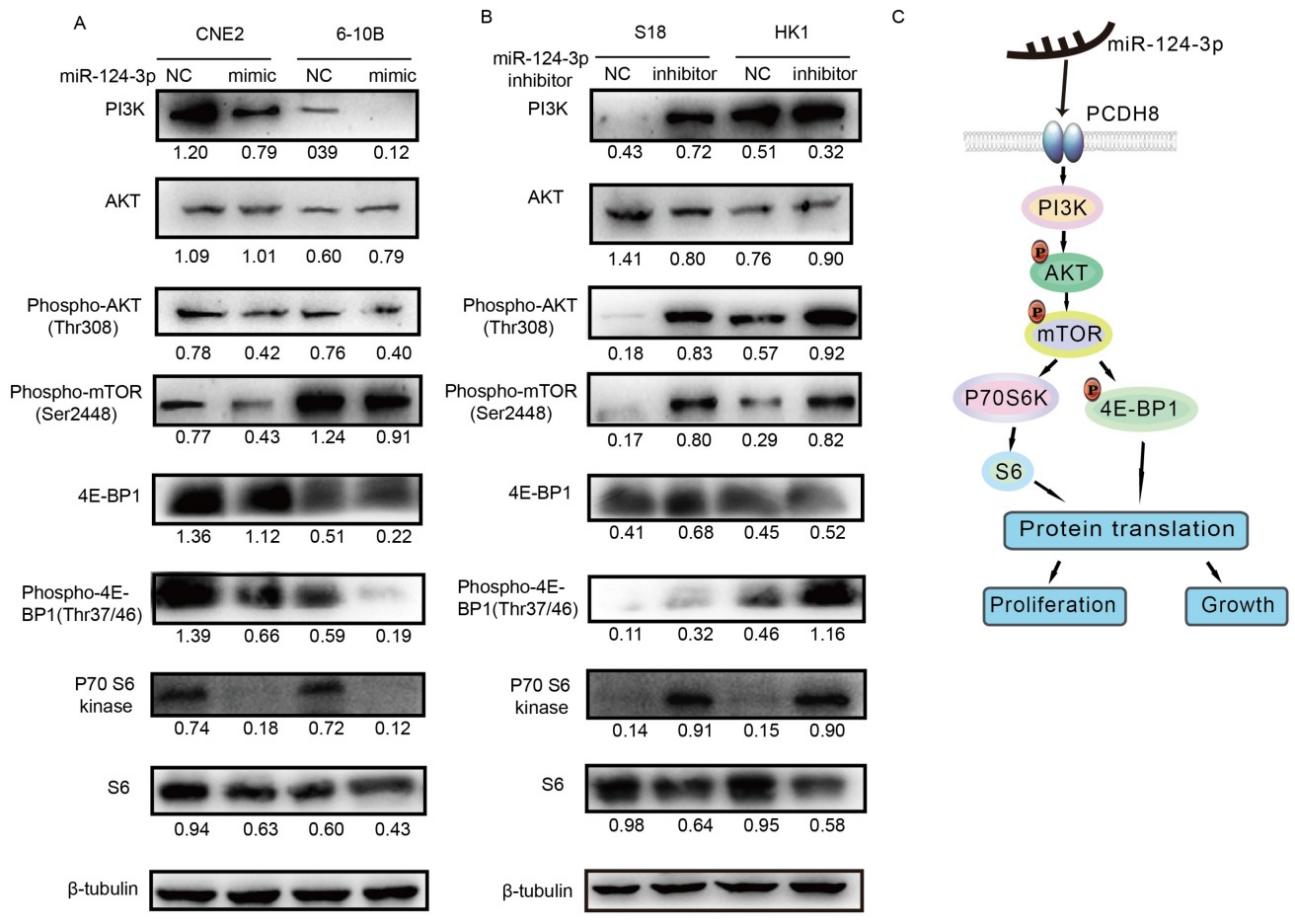
*miR-124-3p* inhibited the proliferation and growth of nasopharyngeal carcinoma cells through activation of the PI3K/AKT/mTOR signaling pathway. (A) Effects of *miR-124-3p* overexpression on the levels of PI3K, phospho-AKT (Thr308), phospho-mTOR (Ser2448), p70 S6 kinase, S6, 4E-BP1, and phospho-4E-BP1 (Thr37/46) in CNE2 and 6-10B cells. (B) Effects of *miR-124-3p* inhibitor on PI3K, phospho-AKT (Thr308), phospho-mTOR (Ser2448), p70 S6 kinase, S6, 4E-BP1, and phospho-4E-BP1 (Thr37/46) levels in S18 and HK1 cells. (C) *miR-124-3p* overexpression decreased activation of the PI3K/AKT/mTOR signaling pathway, thereby inhibiting the proliferation and growth of NPC cells.

Next, we evaluated the protein expression of components of the PI3K/AKT/mTOR signaling pathway, including PI3K, AKT, phospho-AKT, phospho-mTOR, p70 S6 kinase, phospho-p70 S6 kinase, S6, phospho-S6, 4E-BP1, and phospho-4E-BP1, using western blotting to determine whether the signaling pathway was affected by *miR-124-3p*. Notably, PI3K, phospho-AKT (Thr308), phospho-mTOR (Ser2448), p70 S6 kinase, S6, 4E-BP1, and phospho-4E-BP1 (Thr37/46) levels decreased after *miR-124-3p* overexpression in CNE2 and 6-10B cells (Fig. 7A). In contrast, after inhibition of *miR-124-3p* expression in S18 and HK1 cells, PI3K, phospho-AKT (Thr308), phospho-mTOR (Ser2448), p70 S6 kinase, S6, 4E-BP1, and phospho-4E-BP1 (Thr37/46) levels were increased compared with those in the NC group (Fig. 7B). Thus, *miR-124-3p* overexpression decreased activation of the PI3K/AKT/mTOR signaling pathway and thereby inhibited the proliferation and growth of NPC cells (Fig. 7C).

## Discussion

In this study, we found that *miR-124-3p* was significantly downregulated in NPC tissues compared with that in healthy tissues. Moreover, *miR-124-3p* expression inhibited the proliferation and growth of NPC cells *in vitro* and affected the G_1_/S transition. Overall, our findings demonstrated that *miR-124-3p* could inhibit the proliferation and growth of NPC cells by targeting and positively regulating PCDH8.

The abnormal expression of *miR-124* is related to the regulation of important processes in various types of cancer, including cell proliferation, growth, apoptosis, and metastasis. For example, *miR-124-3p* directly targets the 3′-UTR of the mRNA encoding the branched chain amino acid transaminase-1, which suppresses the growth and invasion of esophageal squamous cell carcinoma 20. Similarly, *miR-124-3p* blocks cell migration and invasion by affecting the combined integrin subunit alpha-3 signaling in bladder cancer 35. Furthermore, Luo et al. reported that *miR-124-3p* suppressed the aggressiveness of glioma by targeting Fos-related antigen-2, suggesting potential applications of this miRNA in the treatment of this disease 21. Liu et al. found that abnormal overexpression of *miR-124-3p* in gastric cancer cells could inhibit cell viability, colony formation, and tumor growth *in vivo*; therefore, *miR-124-3p* may act as a potential marker and inhibit tumor growth in gastric cancer 36. Additionally, Idichi et al. showed that *miR-124-3p* expression was low in pancreatic ductal adenocarcinoma tissues and that abnormal expression of *miR-124-3p* suppressed cancer cell migration and invasion in these tissues 37. In our study, we found that *miR-124-3p* expression was low in most NPC cell lines, but overexpression of *miR-124-3p* significantly inhibited proliferation and colony formation in these cell lines. Therefore, *miR-124-3p* may act as a tumor suppressor in NPC.

In a clinical trial, PCDH8 was inhibited in tumor tissues when compared with that in non-tumor tissues, particularly in patients with advanced hypopharyngeal carcinoma, suggesting that PCDH8 could be a prognostic biomarker and target for the treatment of this type of cancer 29. Wang et al. found that PCDH8 was regulated by *miR-217*, which is involved in promoting apoptosis in small cell lung cancer 33. Moreover, Cao et al. also showed that PCDH8 was involved in the metastasis of gastric cancer and that *PCDH8* expression was lower in ovarian cancer tissues than in corresponding adjacent tissues, and contributed to the advanced progression of ovarian cancer 38. Our findings showed that *miR-124-3p* positively regulated *PCDH8* expression through interactions with its 3′-UTR. Moreover, when NPC cells were transfected with a PCDH8 plasmid and then treated with *miR-124-3p* inhibitor, cell proliferation and colony formation decreased compared with that in the control group. These results indicated that overexpression of *PCDH8* could partially rescue the suppressive activity of *miR-124-3p* and that PCDH8 may also act as a tumor suppressor in NPC.

The PI3K/AKT/mTOR signaling pathway is involved in cell proliferation, growth, differentiation, and cytoskeletal reorganization in many types of cancer 39-41. Our study showed that the overexpression of *miR-124-3p* affected tumor progression in NPC cells through the PI3K/AKT/mTOR pathway. When *miR-124-3p* was overexpressed in CNE2 and 6-10B cells, the protein levels of phospho-mTOR, PI3K, p70 S6k, S6, and 4E-BP1 were decreased. These findings suggested that inhibition of NPC cell proliferation and growth by *miR-124-3p* was mediated through the PI3K/AKT/mTOR pathway.

In conclusion, *miR-124-3p* expression was low in NPC tissues and was associated with the tumor suppressor of NPC. Over expression of *miR-124-3p* could decrease the abilities of proliferation and colony formation in NPC cells. Meanwhile, knockdown *miR-124-3p* increased proliferation and colony formation abilities. PCDH8 could partially rescue the proliferation and colony formation role of *miR-124-3p* inhibitor. Our results confirmed that *miR-124-3p* overexpression significantly decreased the proliferation and colony formation of NPC cells by interacting with PCDH8 and inhibiting the activation of the phosphatidylinositol 3-kinase (PI3K)/AKT/mammalian target of rapamycin (mTOR) signaling pathway. Thus, *miR-124-3p* and PCDH8 maybe potential molecular targets that impeded NPC proliferation and colony formation.

## Supplementary Material

Supplementary tables.

## Figures and Tables

**Table 1:** Correlation between mir-124-3p expression and clinicopathological characteristics.

	Variables	Mir-124-3p expression		Total	χ2	p value
		Low	High			
Age (year)					0.044	0.835
	<=45	28	27	55		
	>45	38	34	72		
Stage					2.444	0.118
	I/II	32	38	70		
	III/IV	34	23	57		
	Null					
Cervical lymph node metastasis					0.006	0.94
	No	18	17	35		
	Yes	48	44	92		
	Null					
Sex					1.654	0.198
	Female	13	18	31		
	Male	53	43	96		
	Null					

**Table 2:** Univariate and multivariate analyses of the expression of mir-124-3p correlated with overall survival and recurrence-free survival. (1) The expression of mir-124-3p correlated with overall survival.

Variables	Univariate analysis			Multivariate analysis		
	HR	95%CI	p value	HR	95%CI	p value
Expression	0.303	0.11-0.837	0.021	0.303	0.109-0.842	0.022
Sex	0.863	0.313-2.375	0.775			
Age	1.249	0.51-3.057	0.626			
Stage	8.121	3.763-17.529	<0.001	9.151	4.061-20.623	<0.001

**Table 2A:** Univariate and multivariate analyses of the expression of mir-124-3p correlated with overall survival and recurrence-free survival. (2) The expression of mir-124-3p correlated with recurrence-free survival.

Variables	Univariate analysis			Multivariate analysis		
	HR	95%CI	p value	HR	95%CI	p value
Expression	0.536	0.313-0.92	0.024	0.63	0.366-1.083	0.095
Sex	1.434	0.743-2.769	0.283			
Age	1.31	0.768-2.234	0.321			
Stage	2.504	1.821-3.444	<0.001	2.435	1.765-3.36	<0.001

* Statistically significant (p<0.05)

**Table 3:** Correlation between PCDH8 expression and clinicopathological characteristics.

	Variables	PDH8 expression		Total	χ2	p value
		Low	High			
Age (year)					0.585	0.444
	<=45	28	27	55		
	>45	41	30	71		
Stage					0.412	0.521
	I/II	36	33	69		
	III/IV	33	24	57		
	Null					
Cervical lymph node metastasis					0.75	0.387
	No	17	18	35		
	Yes	52	39	91		
	Null					
Sex					1.042	0.307
	Female	14	16	30		
	Male	55	41	96		
	Null					

**Table 4:** Univariate and multivariate analyses of the expression of PCDH8 correlated with overall survival and recurrence-free survival. (1) The expression of PCDH8 correlated with overall survival.

Variables	Univariate analysis			Multivariate analysis		
	HR	95%CI	p value	HR	95%CI	p value
Expression	0.212	0.062-0.727	0.014	0.311	0.09-1.075	0.065
Sex	1.067	0.354-3.217	0.908			
Age	1.153	0.463-2.867	0.76			
Stage	7.833	3.616-16.968	<0.001	7.146	3.314-15.409	<0.001

**Table 4A:** Univariate and multivariate analyses of the expression of PCDH8 correlated with overall survival and recurrence-free survival. (2) The expression of PCDH8 correlated with recurrence-free survival.

Variables	Univariate analysis			Multivariate analysis		
	HR	95%CI	p value	HR	95%CI	p value
Expression	0.46	0.26-0.813	0.008	0.612	0.342-1.095	0.098
Sex	1.54	0.777-3.051	0.216			
Age	1.285	0.751-2.197	0.36			
Stage	2.505	1.815-3.459	<0.001	2.362	1.704-3.274	<0.001

* Statistically significant (p<0.05)

## Abbreviations

NPC: Nasopharyngeal carcinoma
PCDH8: protocadherin-8
miRNA: microRNA
NC: negative control
