# Supplementary material for: MicroRNA-124-3p inhibited progression of nasopharyngeal carcinoma by interaction with PCDH8 and the inactivation of PI3K/AKT/mTOR pathway
Source: J Cancer. 2021 Jun 11;12(16):4933–44. doi: 10.7150/jca.57152 (PMC8247379; doi:10.7150/jca.57152)
Supplement: Supplementary file 1 — Supplementary tables. [file jcav12p4933s1.pdf]

Table S1. The information of primary antibodies used in western blotting

Target	Supplier	Cat. No.	Application	Dilution
β-tublin	ZEN BIO	200608	WB	1:1000
GAPDH	Sigma-Aldrich	G9545	WB	1:1000
PCDH8	Bioss	bs-11114R	WB	1:1000
cyclinD3	Cell Signaling Technology	2936	WB	1:1000
CDK4	Cell Signaling Technology	12790	WB	1:1000
CDK6	Cell Signaling Technology	3136	WB	1:1000
P21	Cell Signaling Technology	2947	WB	1:1000
P27	Cell Signaling Technology	3686	WB	1:1000
PI3K	ZEN BIO	350172	WB	1:1000
AKT	Cell Signaling Technology	4685	WB	1:1000
Phospho-AKT (Thr308)	Cell Signaling Technology	13038	WB	1:1000
Phospho-mTOR (Ser2448)	Cell Signaling Technology	5536	WB	1:1000
4E-BP1	Cell Signaling Technology	9644	WB	1:1000
Phospho-4E-BP1 (Thr37/46)	Cell Signaling Technology	2855	WB	1:1000
S6	Cell Signaling Technology	2217	WB	1:1000
P70 S6 kinase	Cell Signaling Technology	9202	WB	1:1000

Table S2 The reagents used in ISH

Reagents	Cat. No.	Supplier
Anti-Digoxigenin-AP, Fab fragments	11093274910150U	Roche
Roche DIG Wash and Block Buffer set	11585762001	Roche
microRNA ISH Buffer Set	90000	Exiqon
Proteinase K	P4850	SIGMA
BCIP/NBT Substrate kit	SK-5400	VECTOR Laboratories
